# Sex-specific aortic root anatomy in patients with bicuspid aortic valve undergoing TAVR in a Chinese cohort

**DOI:** 10.1007/s00059-018-4740-0

**Published:** 2018-11-27

**Authors:** F. Du, X. Liu, Q. Zhu, Y. He, J. Jiang, T. Napawan, S. Jaiswal, Z. Chen, J. Wang

**Affiliations:** 1 Department of Cardiology, Second Affiliated Hospital, Zhejiang University School of Medicine, 310009 Hangzhou, Zhejiang China; 2 Department of Clinical Epidemiology and Biostatistics, Second Affiliated Hospital, Zhejiang University School of Medicine, 310009 Hangzhou, Zhejiang China

**Keywords:** Transcatheter aortic valve replacement, Sex ratio, Aortic root, Computed tomography, Aortic valve stenosis, Kathetergestützte Aortenklappenimplantation, Geschlechtsverhältnis, Aortenwurzel, Computertomographie, Aortenklappenstenose

## Abstract

**Objectives:**

The aim of this study is to investigate the sex-specific aortic root anatomy in patients with bicuspid aortic valve (BAV).

**Patients and methods:**

This retrospective study includes 73 consecutive patients with BAV who underwent CT evaluation before transcatheter aortic valve replacement (TAVR) between July 2013 and April 2017 in the Second Affiliated Hospital of Zhejiang University.

**Result:**

The size of the annulus, diameter and height of the sinotubular junction (STJ), height of the coronary artery ostia, and dimension of the aorta were measured. Women had significantly smaller annulus parameters (mean diameter: 23.4 ± 1.8 vs. 26.1 ± 2.1 mm; area: 425.3 ± 59.4 vs. 527.4 ± 84.6 mm^2^; perimeter: 74.3 ± 5.2 vs. 83.2 ± 6.4 mm), and STJ diameter (29.7 ± 3.1 vs. 32.6 ± 4.5 mm) than men (*p* < 0.01 for all), even after adjustment for their smaller body surface area (BSA). Dimension of aorta and height of right coronary artery were also significantly smaller in women, although not when indexing for the BSA. The left ventricular ejection fraction of women is significantly higher than that of men before discharge (60.2 ± 9.7% vs. 53.7 ± 13.6%, *p* = 0.01). There were no differences between women and men in the all-cause 30-day and 1‑year mortality.

**Conclusion:**

Women with BAV had smaller annulus and STJ diameter after indexing for BSA, reflecting a sex-specific difference. There were no differences in 30-day and 1‑year mortality between the two groups.

**Electronic supplementary material:**

The online version of this article (10.1007/s00059-018-4740-0) contains supplementary material, which is available to authorized users.

## Abstract (de)

**Zielsetzung:**

Ziel der vorliegenden Studie war es, die geschlechtsspezifische Anatomie der Aortenwurzel bei Patienten mit bikuspidaler Aortenklappe (BAK) zu untersuchen.

**Patienten und Methoden:**

In die retrospektive Studie wurden 73 konsekutive Patienten mit BAK eingeschlossen, die sich zwischen Juli 2013 und April 2017 im Second Affiliated Hospital of Zhejiang University einer CT-Untersuchung vor kathetergestützter Aortenklappenimplantation („transcatheter aortic valve replacement“ [TAVR]) unterzogen hatten.

**Ergebnisse:**

Die Größe des Anulus, der Durchmesser und die Höhe des sinutubulären Übergangs (STÜ), die Höhe der Koronarostien und die Dimension der Aorta wurden gemessen. Frauen hatten signifikant kleinere Anulusparameter (mittlerer Durchmesser: 23,4 ± 1,8 vs. 26,1 ± 2,1 mm; Fläche: 425,3 ± 59,4 vs. 527,4 ± 84,6 mm^2^; Umfang: 74,3 ± 5,2 vs. 83,2 ± 6,4 mm) und STÜ-Durchmesser (29,7 ± 3,1 vs. 32,6 ± 4,5 mm) als Männer (jeweils *p* < 0,01), auch nach Adjustierung für ihre kleinere Körperoberfläche (KOF). Die Dimension der Aorta und die Höhe der rechten Koronararterie waren ebenfalls bei Frauen signifikant kleiner, allerdings nicht bei Berücksichtigung der KOF. Die linksventrikuläre Ejektionsfraktion vor der Entlassung war bei Frauen signifikant höher als bei Männern (60,2 ± 9,7 % vs. 53,7 ± 13,6 %, *p* = 0,01). Bezüglich der 30-Tages- und 1‑Jahres-Gesamtmortalität bestanden keine Unterschiede zwischen Männern und Frauen.

**Schlussfolgerung:**

Frauen mit BAK hatten einen kleineren Anulus und STÜ-Durchmesser nach Adjustierung für die KOF, worin sich ein geschlechtsspezifischer Unterschied zeigt. Bezüglich der 30-Tages- und 1‑Jahres-Mortalität bestanden keine Unterschiede zwischen den beiden Gruppen.

## Introduction

Transcatheter aortic valve replacement (TAVR) has become an alternative treatment for patients with symptomatic severe aortic valve stenosis (AS) who were deemed as inoperable with high or moderate risk for surgical aortic valve replacement [1]. Patients with a bicuspid aortic valve (BAV) often have concomitant aortopathy such as ascending aortic dilatation, aortic aneurysm and aortic dissection [2]. In China, patients presenting for TAVR have a very high proportion of BAV morphology and severe calcium burden compared with western TAVR registries [3]. BAV has been considered a relative contraindication to TAVR [4]. Recent studies suggest that TAVR appears to be a safe and effective procedure for BAV as well as the tricuspid aortic valve [5–7]. To select appropriate prosthesis size and avoid serious complications such as paravalvular leakage, annulus rupture, coronary obstruction and permanent pacemaker implantation [8–11], 3‑dimensional assessment of aortic root anatomy plays a pivotal role for TAVR [12, 13]. Although several studies showed the sex differences in the aortic root anatomy of patients with a tricuspid aortic valve [14–16], very limited data exist on the impact of sex on aortic root anatomy in patients with BAV undergoing TAVR. In order to investigate the sex differences on the anatomy of aortic root and ascending aorta, we studied a consecutive group of patients with symptomatic severe AS who underwent computed tomography (CT) evaluation as a part of their routine preprocedural evaluation before TAVR.

## Materials and methods

### Study population and aortic root assessment

The study includes 73 consecutive patients with BAV who underwent CT evaluation before TAVR between July 2013 and April 2017 (37 women and 36 men). Patients with a tricuspid aortic valve directly recognized by CT were excluded. After automated reconstruction and segmentation of the aortic root with manual correction, the following planes were identified: annulus plane (virtual circumferential connection of aortic leaflets basal attachments), sinus plane (defined as the plane perpendicular to the centerline that shows the largest cusp dimension), sinotubular junction (STJ) plane (the distal part of the sinus extending upward as the ascending aorta, the junction of sinus and aorta), ascending aortic plane (defined as the plane perpendicular to the long axis of the aortic centerline). We measured the parameters of the annulus plane including maximum diameter, minimum diameter, mean diameter, area and perimeter, area-derived diameter and perimeter-derived diameter were calculated. For the STJ plane, STJ height and average diameter were measured. Coronary height was defined as the distances between the annulus plane and the lower border of the coronary ostia. The maximum diameter and diameter of ascending aorta 4 cm above the annulus plane were measured. The aortic valve calcification degree and aortic root angle were also evaluated. Complications and clinical endpoints were defined according to Valve Academic Research Consortium (VARC) 2 criteria [17]. This study was approved by the institutional review board and carried out according to the principles of the Declaration of Helsinki.

### Statistical analysis

Continuous variables were expressed as mean ± standard deviation (SD). The baseline characteristics of the patients in this study, stratified by sex, were compared using Student *t* test or Mann–Whitney test for continuous variables. The chi-square (χ^2^) test was used for categorical variables. A generalized liner model was used to define the effect of body surface area (BSA) on the studied parameters. In the analysis of covariance, a *P* value <0.05 was defined as statistically significant; also in the other tests the *P* value <0.05 was defined as statistically significant. Statistical analysis was performed with a statistical software package (SPSS, version 19.0, IBM Armonk, NY, USA).

## Results

### Baseline characteristics and CT aortic root data

**Fig. 1:**
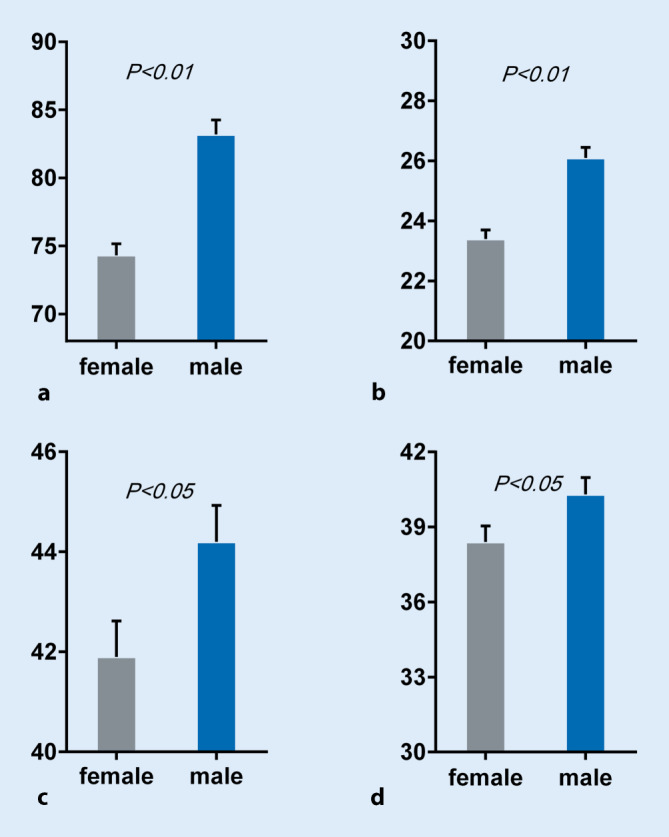
Measurement (in mm) of the annulus and ascending aorta: perimeter (**a**) and mean diameter of annulus (**b**); maximum diameter of ascending aorta (**c**) and ascending aorta diameter 4 cm above the annulus (**d**)

A total of 73 patients with bicuspid AS were treated with TAVR in the Second Affiliated Hospital of Zhejiang University between July 2013 and April 2017. Baseline characteristics of the study population (50.7% women, 49.3% men) are summarized in Table 1. There were no clinical differences including echocardiography data between men and women except that women had a smaller BSA compared to men (1.6 ± 0.1 vs. 1.7 ± 0.2 m^2^, *p* < 0.01). The parameters of aortic root measured by CT are displayed in Table 2 and Fig. 1. In the annulus plane, maximum diameter, minimum diameter, mean diameter, area, perimeter, area-derived diameter and perimeter-derived diameter were significantly smaller in women than in men (*p* <0.01 for all). The average diameter of the STJ was significantly smaller among women than men (29.7 ± 3.1 vs. 32.6 ± 4.5 mm, *p* = 0.003); however, the height of the STJ was comparable among women and men (22.7 ± 3.9 vs. 24.7 ± 5.2 mm, *p* = 0.070). The distance of the annulus plane to the right coronary ostia is smaller in women than in men (15.9 ± 2.4 vs. 17.6 ± 3.6 mm, *p* = 0.021); there was no significant difference in the height of the left coronary ostia in both groups (15.6 ± 2.9 vs. 17.0 ± 4.2 mm, *p* = 0.087). The maximum diameter and the diameter of the ascending aorta 4 cm above the annulus plane were significantly smaller among women than men (41.9 ± 4.4 vs. 44.2 ± 4.4 mm, *p* = 0.032; 38.4 ± 3.9 vs. 40.3 ± 4.1 mm, *p* = 0.040, respectively). There were no significant sex differences in the aortic root angle and aortic valve calcification degree.

**Table 1:** Baseline characteristics and echocardiographic data

Variables	Total (*n* = 73)	Female (*n* = 37)	Male (*n* = 36)	*p* value
*Baseline characteristics*				
Age (year)	74.30 ± 6.15	73.70 ± 5.38	74.92 ± 6.87	0.40
Society of Thoracic Surgery risk of mortality (%)	5.72 ± 3.80	5.83 ± 4.39	5.60 ± 3.14	0.47
Body mass index (kg/m^2^)	22.66 ± 3.03	23.26 ± 3.20	22.04 ± 2.76	0.09
Body surface area (m^2^)	1.65 ± 0.16	1.6 ± 0.1	1.7 ± 0.2	<0.01
Hypertension	36 (49.3%)	16 (43.2%)	20 (55.6%)	0.29
Diabetes mellitus	13 (17.8%)	5 (13.5%)	8 (22.2%)	0.33
Dyslipidemia	25 (34.2%)	15 (40.5%)	10 (27.8%)	0.25
Prior MI	1 (1.4%)	1 (2.7%)	0 (0%)	1.00
Prior PCI	8 (11.0%)	3 (8.1%)	5 (13.9%)	0.68
Prior CABG	0 (0%)	0 (0%)	0 (0%)	–
Prior stroke	1 (1.4%)	0 (0%)	1 (2.8%)	0.49
Peripheral arterial disease	14 (19.2%)	6 (16.2%)	8 (22.2%)	0.52
Atrial fibrillation/flutter	15 (20.5%)	6 (16.2%)	9 (25.0%)	0.35
Prior pacemaker	3 (4.1%)	2 (5.4%)	1 (2.8%)	1.00
NYHA	–	–	–	0.53
NYHA II	13 (17.8%)	6 (16.2%)	7 (19.4%)	–
NYHA III	33 (45.2%)	15 (40.5%)	18 (50.0%)	–
NYHA IV	27 (37.0%)	16 (43.2%)	11 (30.6%)	–
Chronic obstructive pulmonary disease	15 (20.5%)	6 (16.2%)	9 (25.0%)	0.35
Dialysis	1 (1.4%)	0 (0%)	1 (2.8%)	0.49
eGFR (ml/min)	60.85 ± 22.65	63.02 ± 24.32	58.61 ± 20.91	0.41
*Echocardiographic findings*				
Left ventricular ejection fraction (%)	53.80 ± 14.53	55.52 ± 14.91	52.03 ± 14.11	0.31
Aortic valve mean gradient (mm Hg)	60.74 ± 18.17	63.92 ± 18.88	57.47 ± 17.07	0.13
Aortic valve maximum velocity (m/s)	5.00 ± 0.72	5.15 ± 0.70	4.85 ± 0.72	0.08
Aortic valve area (cm^2^)	0.55 ± 0.17	0.54 ± 0.19	0.55 ± 0.14	0.79

*CABG* coronary artery bypass graft, *eGFR* estimated glomerular filtration rate, *MI* myocardial infarction, *PCI* percutaneous coronary intervention, *NYHA* New York Heart Association

**Table 2:** Sex-specific anatomic parameters of aortic root

Parameters	Total (*n* = 73)	Female (*n* = 37)	Male (*n* = 36)	*p* value	General linear model
					*p* value
*Annulus*					
Maximum diameter (mm)	27.6 ± 3.0	25.8 ± 2.1	29.3 ± 2.6	<0.01	<0.01
Minimum diameter (mm)	21.6 ± 2.5	20.7 ± 1.9	22.7 ± 2.7	<0.01	0.01
Mean diameter (mm)	24.7 ± 2.4	23.4 ± 1.8	26.1 ± 2.1	<0.01	<0.01
Area (mm^2^)	475.7 ± 88.8	425.3 ± 59.4	527.4 ± 84.6	<0.01	<0.01
Area-derived diameter (mm)	24.5 ± 2.2	23.2 ± 1.6	25.8 ± 2.1	<0.01	<0.01
Perimeter (mm)	78.7 ± 7.3	74.3 ± 5.2	83.2 ± 6.4	<0.01	<0.01
Perimeter-derived diameter (mm)	25.0 ± 2.3	23.7 ± 1.6	26.5 ± 2.1	<0.01	<0.01
*STJ*					
Diameter (mm)	31.1 ± 4.1	29.7 ± 3.1	32.6 ± 4.5	0.003	0.01
Height (mm)	23.7 ± 4.7	22.7 ± 3.9	24.7 ± 5.2	0.070	0.13
*Ascending aorta*					
Maximum diameter (mm)	43.0 ± 4.5	41.9 ± 4.4	44.2 ± 4.4	0.032	0.14
Diameter (4 cm) (mm)	39.3 ± 4.1	38.4 ± 3.9	40.3 ± 4.1	0.040	0.23
LM height (mm)	16.3 ± 3.7	15.6 ± 2.9	17.0 ± 4.2	0.087	0.12
RCA height (mm)	16.7 ± 3.1	15.9 ± 2.4	17.6 ± 3.6	0.021	0.06
Aortic root angle (°)	54.8 ± 9.6	54.4 ± 10.1	55.3 ± 9.1	0.700	0.91
Aortic root calcification	–	–	–	0.210	–
Mild	4 (5.5%)	3 (8.1%)	1 (2.8%)	–	–
Moderate	11 (15.1%)	8 (21.6%)	3 (8.3%)	–	–
Severe	26 (35.6%)	13 (35.1%)	13 (36.1%)	–	–
Massive	32 (43.8%)	13 (35.1%)	19 (52.8%)	–	–

*LM* left main coronary artery, *RCA* right coronary artery, *STJ* sinotubular junction

### Analysis on the influence of BSA on aortic root

In order to exclude the impact of the BSA on the anatomical structure parameters measured by CT, we consider sex is an important independent variable and BSA as covariance, we found that women have relatively smaller parameters of the aortic annulus including maximum diameter, minimum diameter, mean diameter, area, perimeter, area-derived diameter, perimeter-derived diameter, and the STJ average diameter through the generalized liner model. On the other hand, there were no significant sex differences in the STJ height, coronary ostia height, maximum diameter of aorta, ascending aorta diameter (4 cm), and aortic root angle (Table 2).

### In-hospital complications

According to VARC-2 criteria, there were no differences in perioperative complications between women and men, such as coronary obstruction, life-threatening bleeding, acute kidney injury, new pacemaker implantation and major vascular complication, which are shown in Supplement Table 1. All-cause mortality, moderate to severe paravalvular leakage, stroke and new pacemaker implantation rate at 30-day and 1‑year showed no significant differences in the two groups (Table 3).

**Table 3:** Post-TAVR outcomes

Outcomes	Total	Female	Male	*p* value
*In-hospital (n* *=73)*				
Mortality	3 (4.1%)	2 (5.4%)	1 (2.8%)	1.00
≥Moderate PVL	7 (10.0%)	2 (5.6%)	5 (14.7%)	0.38
Stroke				
Disabling stroke	0 (0%)	0 (0%)	0 (0%)	–
Nondisabling stroke	4 (5.5%)	3 (8.1%)	1 (2.8%)	0.63
New pacemaker implantation	6 (8.3%)	4 (10.8%)	2 (5.7%)	0.72
*30 Day (n* *=73)*				
Mortality	3 (4.1%)	2 (5.4%)	1 (2.8%)	1.00
≥Moderate PVL	3 (4.6%)	1 (3.1%)	2 (6.1%)	1.00
Stroke				
Disabling stroke	0 (0%)	0 (0%)	0 (0%)	–
Nondisabling stroke	5 (6.8%)	4 (10.8%)	1 (2.8%)	0.37
New pacemaker implantation	7 (9.6%)	4 (10.8%)	3 (8.3%)	1.00
*1 Year (n* *=60)*				
Mortality	6 (10.0%)	3 (9.4%)	3 (10.7%)	1.00
≥Moderate PVL	5 (9.6%)	1 (3.4%)	4 (17.4%)	0.22
Stroke				
Disabling stroke	0 (0%)	0 (0%)	0 (0%)	–
Nondisabling stroke	6 (10.0%)	5 (15.6%)	1 (3.6%)	0.26
New pacemaker implantation	7 (11.7%)	4 (12.5%)	3 (10.7%)	1.00

*PVL* paravalvular leakage, *TAVR* transcatheter aortic valve replacement

## Discussion

In order to identify sex differences in the aortic root complex and aorta in patients with BAV undergoing TAVR, we carefully studied and evaluated the anatomy of the aortic root and ascending aorta among patients with a BAV. After adjustment for the BSA, we found that women presented with smaller annulus dimensions such as annulus area, perimeter and diameter, and STJ diameter. However, there were no sex differences in the height of the STJ and coronary ostia, the dimensions of ascending aorta and aortic root angle. Postoperative complications and all-cause mortality at 30-day and 1‑year were similar between women and men. Previous studies have also demonstrated that the annulus, STJ and sinus of Valsalva dimensions and coronary ostia height in patients with a tricuspid aortic valve are larger in men compared with women, whereas dimensions of the ascending aorta are of similar magnitude [14, 16].

BAV disease is the common congenital heart defect. The reported frequency of BAV in western TAVR registries ranges from 1.6% to 6.7% [18, 19], which was much lower compared with those observed in the first TAVR trial in China (47.5%) [3]. BAV has been considered as a relative contraindication because of the high procedural risk related to annulus eccentricity, asymmetrical valve calcification, unequally sized leaflets, and concomitant aortopathy [4]. However, TAVR in symptomatic bicuspid AS was associated with similar procedural complications and prognosis compared with tricuspid AS with the new-generation devices [6].

In our study, women have a smaller annulus. As expected, a smaller aortic annulus is associated with the use of a smaller prosthesis valve. We defined diameter ≤23 mm as small prosthesis valve, and >23 mm as the common prosthesis valve. The proportion of small prosthesis valve used in women was much higher compared with men (≤23 mm 18.9% vs. 0%; >23 mm 81.1% vs. 100%, *p* = 0.01), which is displayed in Supplementary Table 1. A previous study demonstrated that female sex is associated with a smaller annulus and left ventricular outflow tract (LVOT) [14]. In a TAVR meta-analysis by O’Connor et al., the mean aortic annulus diameter was significantly smaller in women [20]. In a single-center study including some Chinese patients with BAV, sex, not BSA was considered an independent predictor of annulus diameter [21].

Women presented with smaller maximum diameter and diameter of ascending aorta 4 cm above annulus plane (41.9 ± 4.4 vs. 44.2 ± 4.4 mm, *p* = 0.032; 38.4 ± 3.9 vs. 40.3 ± 4.1 mm, *p* = 0.040, respectively). These differences did not remain statistically significant after indexing for BSA. There was no annulus rupture in our study, and there was no sex difference in the occurrence of aortic dissection. The dilated ascending aorta increased the risk of aortic rupture and dissection during surgical valve replacement [22]. The position of right coronary artery ostia is lower in women (15.9 ± 2.4 vs.17.6 ± 3.6 mm, *p* = 0.021), but the height of left coronary artery was similar in women and men (15.6 ± 2.9 vs. 17.0 ± 4.2 mm, *p* = 0.087). There were no significant differences in the height of coronary artery after adjustment for BSA in the two groups. Ribeiro et al. discovered that patients with coronary obstruction exhibited a smaller aortic annulus area, sinus of Valsalva (SOV) diameter and STJ diameter, as well as left coronary height. The majority of patients with coronary obstruction were female patients. They also demonstrated that most patients with coronary obstruction had both a left coronary artery (LCA) height <12 mm and a SOV diameter <30 mm [10]. In our study, the mean coronary height of the left main coronary artery (LM) and right coronary artery (RCA) were both greater than 12 mm.

The aortic root angle and aortic valve calcification degree did not differ significantly between women and men in our study. By assessing the calcium volume with CT showed that Chinese people have a threefold excess of leaflet calcium burden compared with western patients, with a leaflet calcium volume of 421 mm versus 142 mm [3]. We also discovered that patients with BAV in our study have severe valve calcification. The proportion of women with moderate and above aortic valve calcification was 91.8%, and 97.2% in men. Recent study data of the pathological morphology suggested that women may have relatively more valvular fibrosis compared with men. Besides aortic valve calcification, fibrosis indeed contributes to the development of valvular stenosis, and as opposed to aortic valve calcification, fibrosis is not detected by CT [23]. It is unknown whether patients with BAV have more valvular fibrosis, which would require valve biopsy for confirmation.

**Fig. 2:**
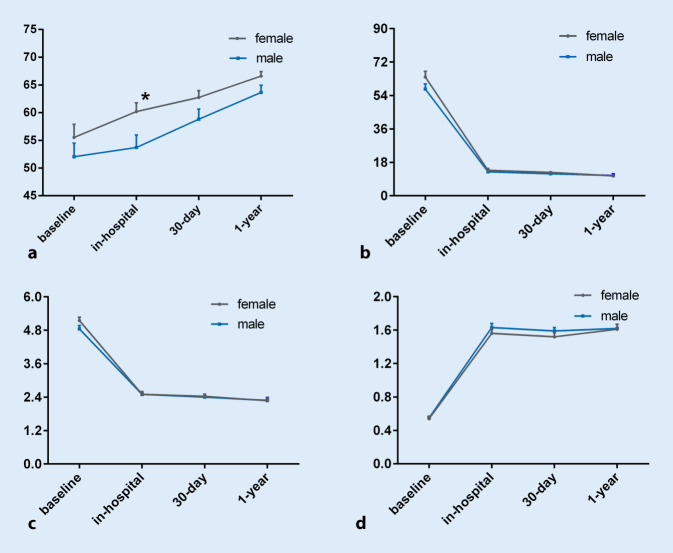
Echocardiographic data. **a** Left ventricular ejection fraction (LVEF, %), **b** mean gradient (mmHg), **c** maximum velocity (m/s) and **d** aortic valve area (cm^2^) across the aortic valve at baseline, in-hospital, 30-day and 1‑year follow-up. ^*^p < 0.05

We found that women have better left ventricular ejection fraction (LVEF) than men after TAVR in the hospital (60.2 ± 9.7% vs. 53.7 ± 13.6%, *p* = 0.01), as shown in Supplement Table 1. But there was no difference in LVEF at 30-days and 1 year between the two groups (Fig. 2). This phenomenon is associated with myocardial response to severe AS. Myocardial remodeling occurs in response to increased left ventricular (LV) afterload and reverse remodeling following correction of severe AS by TAVR or surgery is different in women versus men. Women with severe AS typically manifested more concentric LV geometry, less myocardial fibrosis, and better systolic function compared with men [24, 25]. Surgical studies of patients undergoing aortic valve replacement (AVR) demonstrate less fibrosis on surgical biopsy of myocardium in women, and regression of myocardial hypertrophy is also more rapid in women [26]. Stangl et al. showed that following TAVR, although regression of hypertrophy occurred in men and women, improvement of ejection fraction was significant only in women, thus, potentially reflecting a lower burden of irreversible myocardial damage before TAVR [27]. There is no statistical difference in baseline LVEF between women and men in our study; however, the LVEF of female patients is significantly higher than that of men after TAVR, reflecting better improvement of LVEF in women. The correlation between myocardial remodeling and AS reflects the sex differences in the pathophysiology of myocardial response to the hemodynamic change of AS.

## Study limitation

Limitations should be acknowledged. Our study is a retrospective and single-center study with a small sample. Statistical error in the analysis may be due to the small sample size. Whether the difference of the BAV structure is related to its pathological morphology is unknown.

## Conclusion

Women with BAV have a smaller annulus and STJ diameter, even after indexing for BSA, and thus smaller valve prostheses tend to be selected in clinical practice. There are no differences in all-cause mortality at the 30-day and 1‑year follow-up.

## Caption Electronic Supplementary Material

Supplementary Table 1 In-hospital characteristics
