# Supplementary material for: Sex-specific aortic root anatomy in patients with bicuspid aortic valve undergoing TAVR in a Chinese cohort
Source: Herz. 2018 Nov 27;45(4):375–81. doi: 10.1007/s00059-018-4740-0 (PMC7286940; doi:10.1007/s00059-018-4740-0)
Supplement: Supplementary file 1 — Supplementary Table 1 In-hospital characteristics [file 59_2018_4740_MOESM1_ESM.docx]

**Supplementary Table 1 In-hospital characteristics**

| In-hospital characteristics | Total (n=73) | Female (n=37) | Male (n=36) | p value |
| --- | --- | --- | --- | --- |
| Valve size |  |  |  | 0.01 |
| ≤23 mm | 7 (9.6%) | 7 (18.9%) | 0 (0%) |  |
| >23 mm | 66 (90.4%) | 30 (81.1%) | 36 (100%) |  |
| Mortality | 3 (4.1%) | 2 (5.4%) | 1 (2.8%) | 1.00 |
| MI | 1 (1.4%) | 0 (0%) | 1 (2.8%) | 0.49 |
| Stroke | 4 (5.5%) | 3 (8.1%) | 1 (2.8%) |  |
| Disabling stroke | 0 (0%) | 0 (0%) | 0 (0%) | - |
| Non-disabling stroke | 4 (5.5%) | 3 (8.1%) | 1 (2.8%) | 0.63 |
| Life-threatening bleeding | 8 (11.0%) | 6 (16.2%) | 2 (5.6%) | 0.28 |
| AKI | 3 (4.1%) | 3 (8.1%) | 0 (0%) | 0.25 |
| Major vascular complication | 5 (6.8%) | 2 (5.4%) | 3 (8.3%) | 0.98 |
| Prosthesis-patient mismatch | 18 (25.4%) | 6 (16.7%) | 12 (34.3%) | 0.09 |
| New pacemaker implantation | 6 (8.3%) | 4 (10.8%) | 2 (5.7%) | 0.72 |
| TAV-in-TAV | 7 (9.6%) | 5 (13.5%) | 2 (5.6%) | 0.45 |
| Coronary obstruction | 1 (1.4%) | 1 (2.7%) | 0 (0%) | 1.00 |
| Conversion to open SAVR | 1 (1.4%) | 1 (2.7%) | 0 (0%) | 1.00 |
| Aortic dissection | 3 (4.1%) | 2 (5.4%) | 0 (0%) | 1.00 |
| Echocardiographic findings |  |  |  |  |
| Left ventricular ejection fraction, % | 56.97±12.15 | 60.2±9.7 | 53.7±13.6 | 0.01 |
| Aortic valve mean gradient, mmHg | 13.3±5.2 | 13.7±6.0 | 12.9±4.4 | 0.55 |
| Aortic valve maximum velocity, m/s | 2.5±0.5 | 2.5±0.6 | 2.5±0.4 | 0.99 |
| Aortic valve area, cm^2^ | 1.59±0.30 | 1.56±0.30 | 1.63±0.29 | 0.31 |

***AKI*** acute kidney injury; ***MI*** myocardial infarction; ***SAVR*** surgical aortic valve replacement; ***TAV*** transcatheter aortic valve.
